# A Marine Isolate of *Bacillus pumilus* Secretes a Pumilacidin Active against *Staphylococcus aureus*

**DOI:** 10.3390/md16060180

**Published:** 2018-05-24

**Authors:** Anella Saggese, Rosanna Culurciello, Angela Casillo, Maria Michela Corsaro, Ezio Ricca, Loredana Baccigalupi

**Affiliations:** 1 Department of Biology, Federico II University of Naples, 80126 Naples, Italy; ornellasaggese2010@libero.it (A.S.); rosanna.culurciello@gmail.com (R.C.); ericca@unina.it (E.R.); 2 Department of Chemical Sciences, Federico II University of Naples, 80126 Naples, Italy; angela.casillo@unina.it (A.C.); mariamichela.corsaro@unina.it (M.M.C.)

**Keywords:** SF214, *B. pumilus*, antimicrobials, lipopeptides, surfactants, surfactin, pumilacidin

## Abstract

Producing antimicrobials is a common adaptive behavior shared by many microorganisms, including marine bacteria. We report that SF214, a marine-isolated strain of *Bacillus pumilus*, produces at least two different molecules with antibacterial activity: a molecule smaller than 3 kDa active against *Staphylococcus aureus* and a molecule larger than 10 kDa active against *Listeria monocytogenes.* We focused our attention on the anti-*Staphylococcus* molecule and found that it was active at a wide range of pH conditions and that its secretion was dependent on the growth phase, medium, and temperature. A mass spectrometry analysis of the size-fractionated supernatant of SF214 identified the small anti-*Staphylococcus* molecule as a pumilacidin, a nonribosomally synthesized biosurfactant composed of a mixture of cyclic heptapeptides linked to fatty acids of variable length. The analysis of the SF214 genome revealed the presence of a gene cluster similar to the *srfA-sfp* locus encoding the multimodular, nonribosomal peptide synthases found in other surfactant-producing bacilli. However, the *srfA-sfp* cluster of SF214 differed from that present in other surfactant-producing strains of *B. pumilus* by the presence of an insertion element previously found only in strains of *B. safensis*.

## 1. Introduction

The emergence of pathogenic antibiotic-resistant bacteria urges for an increased attention to the identification and characterization of new antimicrobials. Marine organisms have remarkably contributed to the discovery and production of novel antimicrobials, and in general, of biomolecules of pharmaceutical relevance. Indeed, over 50% of all drugs approved in the U.S. between 1981 and 2002 are based on either marine bioactive compounds or their synthetic analogs [1]. However, for its enormous biodiversity, the marine environment is still considered a largely unexplored source of antimicrobial agents [1,2]. Sponges, marine algae, and bacteria all produce a large variety of secondary metabolites of remarkable chemical diversity with potential antimicrobial activity [2]. In recent years, terpenoids, steroids, phenolic compounds, alkaloids, polyketides, and peptides produced by marine organisms have shown antibacterial, antiviral, and antifungal activities [2]. Bacterial peptides and lipopeptides with antimicrobial activity are probably the most promising molecules for future biomedical applications because of their relative ease of overproduction and purification. Over the years, several examples of peptides and lipopeptides produced by marine bacteria and showing antimicrobial activities have been reported. These include the cyclic lipopeptides maribasins A and B, produced by *Bacillus marinus* and active against several phytopathogens and fungi [3]; the lipopeptide tauramamide, produced by *Brevibacillus laterosporus* and selectively active against *Enterococcus* cells [4]; and a thiopeptide produced by *Nocardiopsis* sp. TP-1161 and active against a wide range of bacteria [5]. Often, the synthesis of antimicrobial peptides and lipopeptides does not rely on the translational machinery, but on multimodular enzymes, the nonribosomal peptide synthases (NRPS) [6]. These enzymatically produced peptides are biosurfactants with antimicrobial activity and often contain cyclic structures, D-amino acids, and various modifications such as *N*-methyl and *N*-formyl groups or glycosylation, acylation, or hydroxylation [7]. The peptide moiety of these biosurfactants is generally formed by 7–15 residues (hydrophilic head) and is bound to a fatty acid (hydrophobic tail) of variable lengths [8,9,10]. Various species of the *Bacillus* genus produce similar nonribosomal lipopeptides grouped in the surfactin, iturin, or fengycin families [11]. Members of the surfactin family are surfactin, produced by strains of *B. subtilis* and *B. amyloliquefaciens*; lichenysin, produced by strains of *B. licheniformis*; and pumilacidin, produced by strains of *B. pumilus* and *B. safensis* [12]. The best characterized of these is surfactin, a mixture of cyclic lipopeptides formed by a heptapeptide with the sequence Glu–(Leu/Ile/Val)–Leu–(Leu/Ile/Val)–Asp–Leu–(Leu/Ile/Val) and a beta-hydroxy fatty acid with chains of length ranging from 13 to 16 carbons and differing mostly in the fatty acid substitutions [12]. While surfactins have a wide spectrum of activity against various Gram-positive and Gram-negative bacteria [13,14], pumilacidins have antiviral [15] and antimotility [16] activities, but no data are currently available on their antibacterial activity.

We screened a collection of marine-isolated bacteria, all Gram-positive, aerobic endospore formers belonging to the *Bacillus* genus, to search for strains producing molecules with antimicrobial activity against a panel of target bacteria. The only strain in our screen that showed antibacterial activity was *Bacillus pumilus* SF214, a previously characterized strain, shown to produce a water-soluble yellow-orange pigment [17]. The still-uncharacterized pigment is essential to protect SF214 cells against oxidative stress and its synthesis is highly regulated, with a maximum of production in minimal media at 25 °C and during the stationary phase of growth [18,19]. The aim of this work was then to provide a characterization of the antibacterial molecules produced and secreted by SF214 and of the genes involved in their biosynthesis.

## 2. Results and Discussion

### 2.1. SF214 Secretes Two Antimicrobials Active Against Staphylococcus aureus or Listeria monocytogenes

**Figure 1:**
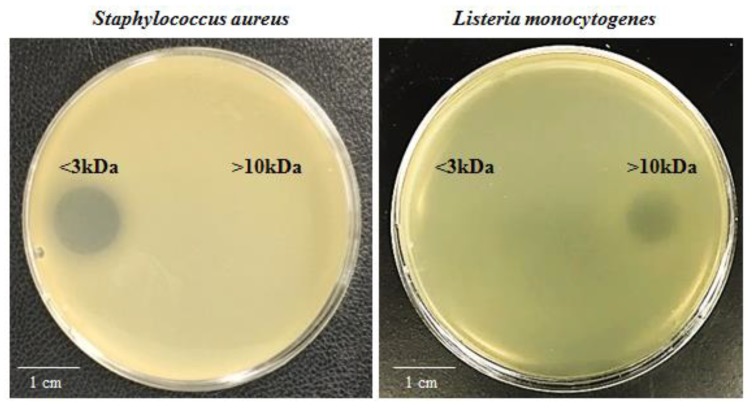
Antimicrobial plate assays. Fractions of *Bacillus pumilus* SF214 supernatant were tested against target bacteria. The fraction containing molecules smaller than 3 kDa was active against *S. aureus* and not against *L. monocytogenes*, while the fraction containing molecules larger than 10 kDa was active against *L. monocytogenes* and not against *S. aureus*.

In order to identify new molecules with antimicrobial activity, we used a collection of fifteen *Bacillus* strains all isolated from marine samples (Supplementary Materials Table S1). All strains were grown for 16 h at 30 °C in LB (Luria-Bertani) medium, the culture supernatants were filter-sterilized, and 10 μL of each supernatant was used for antimicrobial plate assays with a panel of target bacterial strains. Only one of the fifteen strains tested, SF214, showed antimicrobial activity against *Staphylococcus aureus* and *Listeria monocytogenes* (Supplementary Materials Table S2). To characterize the antimicrobial activity of SF214, the supernatant was size-fractionated and the various fractions tested in antimicrobial plate assays against the two sensitive target bacteria. While the molecule active against *S. aureus* was in the fraction smaller than 3 kDa, the one active against *L. monocytogenes* was in the fraction of molecules larger than 10 kDa (Figure 1), indicating that SF214 produces and secretes at least two different molecules with antimicrobial activity.

### 2.2. Production of Both Antimicrobials Is Strictly Regulated

SF214 was grown at various temperatures and in different growth media. The supernatants of the various growth experiments were size-fractionated and the fractions smaller than 3 kDa and larger than 10 kDa tested against *S. aureus* and *L. monocytogenes,* respectively. As shown in Table 1, the anti-*Staphylococcus* activity was stronger in supernatants obtained with minimal (S7) medium than in rich (LB or BHI, Brain Heart Infusion) or in sporulation-inducing (DS, Difco Sporulation) media and was stronger in supernatants of cell growth at 25 °C than at 30 or 37 °C. The anti-*Listeria* activity was similarly strong in supernatants obtained with BHI and S7 media, while it was slightly less strong in LB or DS media. In the minimal S7 medium, the anti-*Listeria* activity was similarly strong at 25 °C and 30 °C and slightly less strong at 37 °C (Table 1).

**Figure 2:**
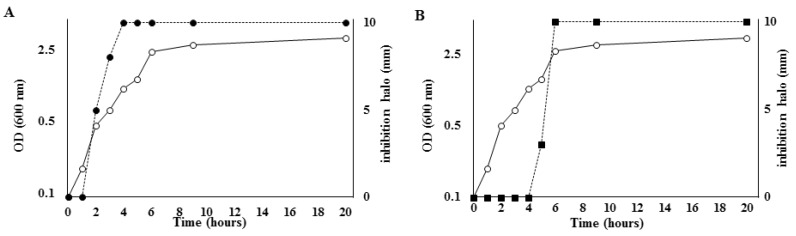
Production of the antimicrobials during growth. SF214 was grown in minimal (S7) medium at 25 °C and the growth curve reported (white symbols). Aliquots of the cell culture were collected at various times, size-fractionated, and tested against the target bacteria. Dashed lines indicate the antimicrobial activity (diameter in mm of inhibition halo) of the fraction larger than 10 kDa against *L. monocytogenes* (**A**) and of the fraction smaller than 3 kDa against *S. aureus* (**B**).

Production of the two antimicrobial molecules was also analyzed during growth in S7 medium at 25 °C. As shown in Figure 2, while the anti-*Listeria* molecule was produced during the exponential and stationary growth phases and the increase of activity paralleled the increase in the number of cells, production of the anti-*Staphylococcus* molecule only started in the late stationary growth phase and continued during the stationary phase, revealing the typical pattern of synthesis of secondary metabolites. The production profile of the antimicrobial smaller than 3 kDa resembled that of the pigment produced by SF214 cells. Indeed, the pigment was also produced in the late stationary phase of growth, preferentially at 25 °C in minimal (S7) medium [17]. However, single mutants of SF214 that did not produce [18] or produced a larger amount [19] of pigment showed an anti-*Staphylococcus* activity identical to that of the wild-type strain (data not shown), indicating that the pigment and the antimicrobial molecules were independent molecules.

Results of Table 1 and Figure 2 thus suggest that SF214 produces a secondary metabolite, smaller than 3 kDa and active against *S. aureus* and a product of the primary metabolism, larger than 10 kDa and active against *L. monocytogenes.* We decided to focus our attention on the secondary metabolite active against *Staphylococcus aureus* for all further experiments.

### 2.3. The Anti-Staphylococcus Molecule Was Stable to Heat, pH, and Treatments with Chemicals and Enzymes

To obtain a preliminary characterization of the antimicrobial molecule, we analyzed its stability in a wide range of pH and temperature conditions and after treatments with chemicals and enzymes. As reported in Table 2, the molecule was similarly active against its target cells after 1 or 5 h of incubation at pH values ranging from 4.0 to 13.0 and was only slightly reduced at pH 2.0. The antimicrobial was fully active after 15 min of incubation at 60 or 80 °C and the activity was only reduced after 15 min at 100 °C (Table 3). None of the organic solvents or enzymes tested showed any effect on the antimicrobial activity of the small anti-*Staphylococcus* molecule (Table 3).

### 2.4. Purification and Chemical Characterization of the Anti-*Staphylococcus* Molecule

**Figure 3:**
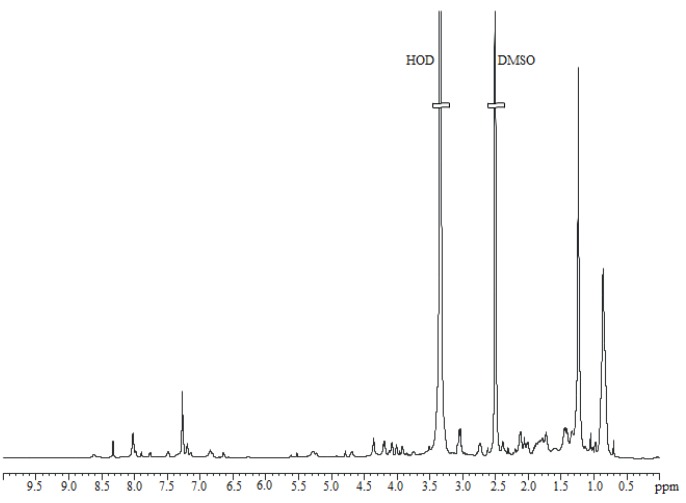
NMR spectrum. ^1^H NMR spectrum of the active fraction, recorded at 298 K, in DMSO, at 600 MHz.

The supernatant fraction active against *S. aureus* was extracted with chloroform. Both aqueous and organic phases were tested for antimicrobial activity against *S. aureus*, revealing that the organic phase was endowed with the antimicrobial activity. The ^1^H NMR spectrum suggested the presence of a complex mixture of compounds (data not shown). To investigate the nature of the antimicrobial molecule, we performed a preliminary purification of the bioactive compound by using a C18 reverse phase column and eluted with acetonitrile and water in different ratios. The obtained fractions were tested to evaluate the antimicrobial activity on *S. aureus*. The results clearly indicated that the active fraction was eluted with the 50% acetonitrile solution. The ^1^H NMR spectrum of the active fraction (Figure 3) suggested the presence of long hydrocarbon chains, due to the intense signals in the range of 0.5–1.5 ppm. In addition, signals appearing between 7.0 and 8.5 ppm strongly suggested N–H protons, that together with signals around 4 ppm, indicated a peptide backbone (Figure 3) [9,15].

**Figure 4:**
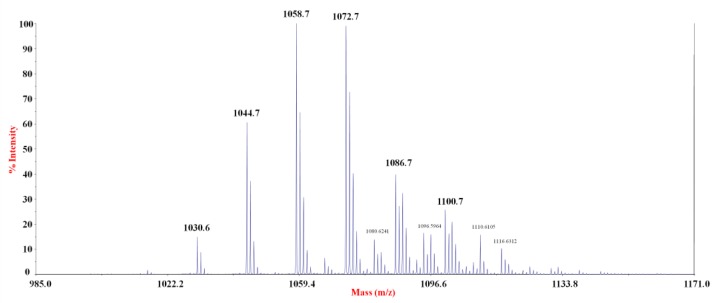
Positive ions MALDI-TOF mass spectrum of the active fraction.

To test if the compounds were lipopeptides, a positive ion mass spectrometry experiment was performed by a MALDI-TOF instrument. The spectrum indicated the presence of several clusters of signals, within the range of *m*/*z* 1000–1200. By comparing the *m*/*z* values with the mass numbers reported for the lipopeptides commonly produced from *Bacillus* strains, we found that the signals at *m*/*z* 1030.6, 1044.7, 1058.7, and 1072.7 were attributable to the sodium adduct of pumilacidins (Figure 4, Table 4) [15].

**Figure 5:**
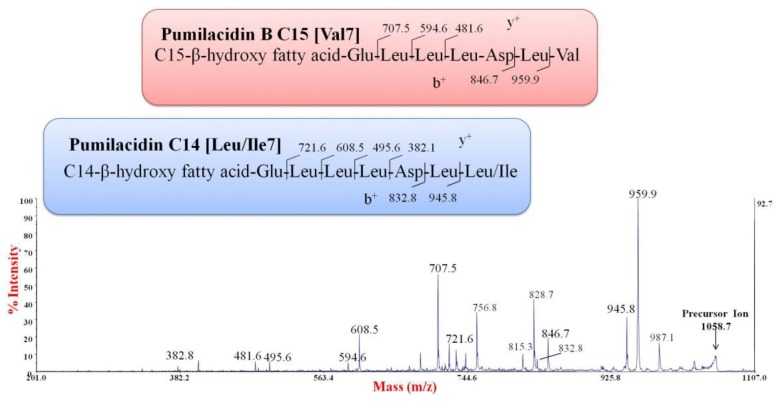
Positive ion MALDI-TOF MS/MS spectrum of the pumilacidin precursor ion at *m*/*z* 1058.7. b^+^ and y^+^ fragment ions are reported for pumilacidin isoforms in the insert.

Since some pumilacidin isoforms have the same *m*/*z* values, MS/MS analyses were necessary to identify the amino acid sequence of the peptide portion [15,21,22]. The experiments were carried out on the most abundant signals of the mass spectrum. As an example, Figure 5 displays the positive ion MS/MS spectrum obtained from the precursor ion at *m*/*z* 1058.7. Through this experiment, it was possible to identify two series of b^+^ fragment ions at *m*/*z* 959.9–846.7 and *m*/*z* 945.8–832.8, indicating that at least two isoforms with Val or Leu/Ile at the C terminus, respectively, were present (Figure 5). This was confirmed by the y^+^ fragment ions, since two different series at *m*/*z* 707.5–594.6–481.6 and *m*/*z* 721.6–608.5–495.6 were found (Figure 5). These fragmentation patterns suggested that the signal at *m*/*z* 1058.7 corresponded to the B isoform, with a C15(3OH) [20]; and to another isoform with a C14(3OH). Similarly, the fragmentation patterns of parent ions at *m*/*z* 1044.7 and *m*/*z* 1072.7 indicated the same amino acid sequences, with different hydroxylated fatty acids (Table 4). Since the signals at *m*/*z* 1030.6, 1086.7, and 1100.7 displayed a low intensity, the corresponding structures reported in Table 4 were suggested by analogy. Some of these pumilacidins have been already characterized [15], as reported in Table 4. However, as far as we know, the isoforms containing 3-hydroxy-tridecanoic, -tetradecanoic, and -octadecanoic fatty acids have not been reported yet.

In conclusion, the NMR and MALDI-TOF MS/MS spectra indicated that the anti-*Staphylococcus* activity was due to a mixture of lipopeptides with structures similar to those of pumilacidins produced by strains of *B. pumilus* and *B. safensis* [12,15]. SF214 produced two isoforms of pumilacidin corresponding in their heptapeptide sequences to the previously reported pumilacidins [15] and with hydrophobic chains ranging in size between 13 and 18 carbon atoms (Table 4).

### 2.5. The srfA-sfp Locus of SF214

To characterize the pumilacidin biosynthetic pathway in SF214, we searched the SF214 genome for homologs of genes coding for the NRPS responsible for the nonribosomal synthesis of surfactants in bacilli. A *srfA-sfp* locus was identified in SF214, and the various genes of the locus showed a high similarity (over 90%) with homologs of other pumilacidin-producing strains of the *B. pumilus* and *B. safensis* species (Table 5).

**Figure 6:**
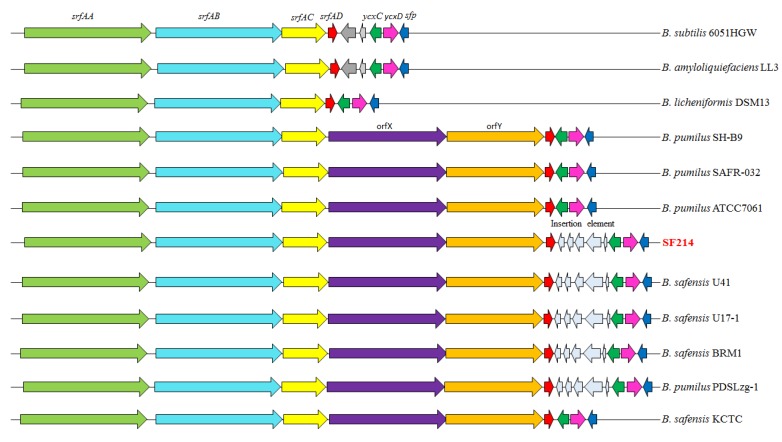
The *srfA-sfp* locus of surfactant-producing bacilli. Arrows of the same color indicate orthologous genes.

The *srfA* operon (Figure 6) was organized in six genes: *srfAA, srfAB,* and *srfAC,* coding for the surfactin synthase subunits 1, 2, and 3 respectively; and *srfAD*, coding for a surfactin synthase thioesterase subunit and two still-uncharacterized genes, *orfX* and *orfY*. These two genes are not present in surfactin- or lychenisin-producing species [12] and are only found in strains of *B. pumilus* and *B. safensis* that produce pumilacidins [12]. The presence of *orfX*/*orfY* is the main difference between the *srfA* operon of the *B. pumilus*/*B. safensis* and that of the *B. subtilis*/*B. amyloliquefaciens*/*B. licheniformis* group of surfactant-producing bacilli [12].

**Figure 7:**
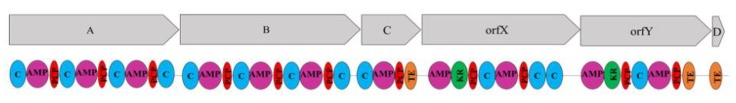
The *srfA* operon of SF214 and its modular products. The prediction diagram was obtained by using the HMMER tool (https://www.ebi.ac.uk/Tools/hmmer/) [23]. The same colors indicate the same module.

All six genes of the srfA operon of SF214 coded for putative proteins with the typical modular organization of NRPS (Figure 7). In particular, srfAA-AC genes coded for proteins arranged in modules of 10, 10, and 4 domains, respectively. In each module of SrfAA and SrfAB of SF214, a condensation domain (C) was followed by an AMP-binding (AMP) and a peptidyl carrier (PCP) domain. The tenth domain of both proteins of SF214 was an additional C domain (Figure 7), while in other pumilacidin-producing bacteria, an epimerization (E) domain was present at the C-terminal part of SrfAA and SrfAB [12]. SrfAC of SF214 had four domains, as in other pumilacidin-producing strains (Figure 7). OrfX and OrfY were characterized by eight and seven domains, respectively. In SF214, the seventh domain of OrfX was a C domain and not an E domain, as in other strains that produce pumilacidins [12]; while OrfY and SrfAD showed the same organization as that of other *B. pumilus* or *B. safensis* strains.

Like in other lychenysin- or pumilacidin-producing bacteria, in SF214, the *yxc* operon is formed by only two genes, *ycxC* and *ycxD*, and homologs of *ycxAB* genes of *B. subtilis* and *B. amyloliquefaciens* are not found (Figure 6).

With respect to the group of pumilacidin producers, an additional difference was observed in SF214. It contained an insertion element between *srfAD* and *ycxC*, more commonly found in *B. safensis* than in *B. pumilus* strains [12]; (Figure 6). The insertion element of SF214 coded for five putative products, highly similar (84–87%) to those encoded by *B. safensis* strains (Table 5).

**Figure 8:**
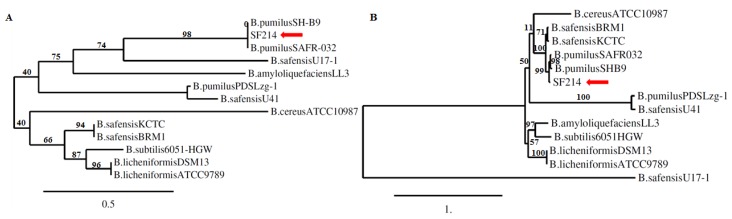
Phylogenetic trees. Diagrams were obtained on the basis of 16S rRNA (**A**) and gyrase B subunit (**B**) gene sequences (http://www.phylogeny.fr/phylogeny.cgi) [24].

The presence of such an insertion element and the high similarity between the *B. pumilus* and *B. safensis* species raised the possibility that SF214 was originally [17] misclassified as *B. pumilus.* To address this point, we performed an accurate phylogenetic analysis based on the 16S RNA and *gyrB* gene sequences. In both cases, the SF214 sequences shared the highest similarity with sequences of *B. pumilus* strains (Figure 8), suggesting SF214 as being an isolate of *B. pumilus.* To confirm this finding, the entire genome of *B. pumilus* SF214 was compared with the genomes of *B. pumilus* SAFR-032, *B. safensis* KCTC, and *B. safensis* U17. The average nucleotide identity (ANI) values, reported in parentheses, indicated that SF214 is more similar to *B. pumilus* SAFR-032 (94.87%) than to *B. safensis* KCTC (92.44%) and *B. safensis* U17 (92.40%).

## 3. Experimental Section

### 3.1. Bacterial Strains

*Bacillus* strains are listed in Table S1 (Supplementary Materials). The antibacterial activity of these strains was evaluated using a panel of indicator strains: six Gram-positive (*Streptococcus faecalis*, *Staphylococcus aureus*, *Bacillus megaterium*, *Listeria monocytogenes*, *Mycobacterium smegmatis*, and *Enterococcus faecalis*) and five Gram-negative (*Escherichia coli*, *Salmonella enterica typhimurium*, *Pseudomonas fluorescens*, *Shigella sonnei*, and *Citrobacter freundii*) bacteria [25]. All bacteria were grown in LB broth (8 g/L NaCl, 10 g/L tryptone, 5 g/L yeast extract) aerobically at 25 °C.

To characterize the antimicrobial production of SF214, the strain was grown at 37 °C in different media, with a base of DSM (Difco sporulation medium: bacto nutrient broth 8 g/L, KCl 1 g/L, and MgSO_4_ 0.25 g/L, sterilized at 121 °C for 30 min). To 1 L of this solution, 1 mL of each of the following filter-sterilized solutions were added: 1 M Ca(NO_3_)_2_, 10 mM MnCl_2_ and 1 mM FeSO_4_; S7 (50 mM morpholinepropanesulfonic acid (MOPS) (adjusted to pH 7.0 with KOH), 10 mM (NH_4_)_2_SO_4_, 5 mM potassium phosphate (pH 7.0), 2 mM MgCl_2_, 0.9 mM CaCl_2_, 50 μM MnCl_2_, 5 μM FeCl_3_, 10 μM ZnCl_2_, 2 μM thiamine hydrochloride, 20 mM sodium glutamate, 1% glucose, 0.1 mg/mL phenylalanine, and 0.1 mg/mL tryptophan); and brain heart infusion (BHI: beef heart 5 g/L, calf brains 12.5 g/L, Na_2_HPO_4_ 2.5 g/L, glucose 2 g/L, peptone 10 g/L, and NaCl 5 g/L).

### 3.2. Filter-Sterilized and Size-Fractionated Supernatants

*Bacillus pumilus* SF214 [17] was grown in LB broth for 24 h at 37 °C; the culture was diluted and used to inoculate fresh LB, Difco Sporulation (DS), or S7 media. Cells of SF214 were then grown for 16 h at 25 °C, where not otherwise specified. The culture was centrifuged (1000× *g* for 10 min at Room Temperature) and the supernatant filter-sterilized with a 0.22-µm filter (Millipore, Bedford, MA, USA). The supernatants were size-fractionated (3-kDa and 10-kDa cutoff spin column; Centricon, Millipore). Fractions were tested for antimicrobial activity and reported as the diameter (in millimeters) of the inhibition halo in plate assays.

### 3.3. Antimicrobial Plate Assay

Antimicrobial activity was determined with the method described by Schillinger and Lüeke [26] with the following modifications: 10 µL of each bacterial culture (strains listed in Table 1) in stationary growth phase were spotted on the surface of a sterile LB agar plate and the spots air-dried. 100 µL of an exponential culture of each of the indicator bacterial strains was mixed with 10 mL of soft agar (0.7%) and poured over the plate. The plates were incubated aerobically overnight at 37 °C and the inhibition halos were measured and reported in mm.

### 3.4. Stability of Antimicrobials at Different pH, Temperature, Chemical, and Enzyme Conditions

Enzymes (100 μg/mL) and 10% organic solvents (see Table 3) were added to 100 µL of culture supernatant. Enzyme-treated samples were incubated 1 h at 37 °C (42 °C in the case of proteinase K) and solvent-treated samples were incubated for 1 and 5 h at 25 °C, and subsequently, 10 µL aliquots were tested for antimicrobial activity as described above. The effects of pH and heat (Table 2 and Table 3) on supernatants were analyzed by assaying the antimicrobial activity after 15 min of incubation at 60, 80, and 100 °C and after 1- and 5-h incubations at 30 °C in 50 mM phosphate buffer (pH 6.0), adjusted to the various pH with HCl and NaOH.

### 3.5. Lipopeptide Purification and Preliminary Analysis

The fraction active against *S. aureus* was extracted three times with chloroform (v/v 1:1). The organic phases were combined and concentrated in a rotary vacuum evaporator to obtain 18 mg. The organic phase was fractionated on a C18 reverse phase column (Sigma, Aldrich, Italy, 30 mL, 40 cm × 0.5 cm, fraction volume 4 mL) and eluted with CH_3_CN/H_2_O, ranging from 10% to 100% of CH_3_CN. The collected fractions were tested for antimicrobial activity and the active fraction, eluted with 50% CH_3_CN, was preliminary analyzed by ^1^H NMR. The sample was dissolved in 0.5 mL of DMSO-*d*_6_, and the ^1^H NMR spectrum was recorded at 298 K using a BrukerAvance 600 MHz spectrometer equipped with a cryoprobe (Bruker Italia, Milano, Italy).

### 3.6. Characterization of Lipopeptide

MALDI-TOF spectra were acquired in reflector positive mode by using a SCIEX TOF/TOF™ 5800. Fractions were dissolved in CHCl_3_/CH_3_OH (v/v 1:1) at a concentration of 1.0 mg/mL, and 0.5 μL of the solution was mixed on the plate with 0.5 μL of a 20 mg/mL solution of 2,5-dihydroxy benzoic acid in CH_3_CN/TFA 0.1 (v/v 7:3). The *m*/*z* values were measured in the range from *m*/*z* 500 to 5000.

MALDI TOF-MS/MS coupled with CID (Collision-Induced Dissociation) was used to analyze the fragment ions of lipopeptides for further characterization of the amino acid sequence.

### 3.7. Bioinformatic Analysis

The genome of SF214 (PRJNA290581) was analyzed by searching for homologs of the *srfA-sfp* locus of other biosurfactant-producing strains of the *Bacillus* spp. by using a BLAST analysis (http://blast.ncbi.nlm.nih.gov/) [27]. The prediction of the protein structure for the products of the *srfA* operon of SF214 was obtained by using the web server HMMER (https://www.ebi.ac.uk/Tools/hmmer/) [23]. For the phylogenetic analysis, the complete 16S ribosomal RNA (rRNA) and gyrase subunit B (*gyrB*) gene sequences of SF214 and of closely related type strains of *Bacillus* species (from GenBank database) were used to obtain two phylogenetic trees by using the web service “Phylogeny.fr” [24]. The genome comparison and the analysis of ANI values were performed using Jspecies software [28,29].

## 4. Conclusions

We report that a marine-isolated strain of *B. pumilus*, SF214, produces and secretes two molecules with antimicrobial activity: a molecule smaller than 3 kDa active against *Staphylococcus aureus* and a molecule larger than 10 kDa active against *Listeria monocytogenes.* The anti-*Staphylococcus* molecule showed the typical expression pattern of secondary metabolites and we focused our attention on the characterization of this small molecule. A mass spectrometry analysis identified the anti-*Staphylococcus* molecule as a mixture of lipopeptides formed by a cyclic heptapeptide with the sequence Glu-Leu-Leu-Leu-Asp-Leu-[(Leu/Ile)/Val] and a fatty acid with carbon chains variable in length between 13 and 18 atoms. A similar structure is shared by biosurfactants of the surfactin family [11]. Molecules of this family are nonribosomally synthesized and classified as surfactins, produced by strains of *B. subtilis* and *B. amyloliquefaciens*; lichenysins, produced by strains of *B. licheniformis*; and pumilacidins, produced by strains of *B. pumilus* and *B. safensis* [12]. These molecules all have very similar structures, with the heptapeptides sharing similar amino acid sequences and the fatty acids having carbon chains of variable length (C12–C18) [12,20]. The surfactant produced by SF214 has hydrophobic chains of highly variable length (C13–C18) and differs from pumilacidins produced by other *B. pumilus* strains that are characterized by less variability, with chains of C12–C14 [30] or C15–C17 [31].

Additional differences between SF214 and other pumilacidin-producing strains of *B. pumilus* are in the organization of the *sfrA-spf* locus coding for the multimodular enzyme responsible for the nonribosomal synthesis of the biosurfactant. The SrfA and SrfB proteins are organized in 10 modules, but the epimerization domain present at the C-terminal end of both proteins of other pumilacidic-producing strains is replaced in SF214 by a condensation domain. Moreover, an insertion element of 4684 bp, previously identified in members of the *B. safensis* species, is present in SF214 between *srfAD* and *ycxC*.

Understanding whether and how the differences observed in the *srfA-sfp* locus cause the minor differences observed in the structure of the pumilacidin of SF214 with respect to those of other strains of *B. pumilus* and *B. safensis* will be a challenging future perspective opened by this report.

## Figures and Tables

**Table 1:** Production of antimicrobials at various growth conditions.

Medium	Temperature (°C)	Supernatant Activity ^a^ > 10 kDa against *L. monocytogenes*	Supernatant Activity ^a^ < 3 kDa against *S. aureus*
LB (Luria-Bertani)	25	7	5
BHI (Brain Heart Infusion)	25	10	5
DS (Difco Sporulation)	25	7	5
S7	25	10	10
S7	30	10	7
S7	37	5	5

BHI broth was supplemented with 0.1% glucose. ^a^ Diameter (mm) of the inhibition halo in plate assays.

**Table 2:** Effect of pH on antimicrobial activity.

pH	Supernatant Activity ^a^ < 3 kDa against *S. aureus*	
	1 h	5 h
2	7	7
4	10	10
7	10	10
10	10	10
13	10	10

^a^ Diameter (mm) of the inhibition halo in plate assays.

**Table 3:** Effects of heat, chemicals, and enzymes on antimicrobial activity.

Treatment	Supernatant Activity ^b^ < 3 kDa against *S. aureus*
None	10
Trypsin ^a^	10
Proteinase K ^a^	10
DNase ^a^	10
Ribonuclease A ^a^	10
Acetone ^c^	10
Ethyl alcohol ^c^	10
Chloroform ^c^	10
Toluene ^c^	10
Incubation (15 min) at:	
60 °C	10
80 °C	10
100 °C	5

^a^ The enzyme concentration was 100 μg/mL [20]. ^b^ Diameter (mm) of the inhibition halo in plate assays. ^c^ A 10% (v/v) concentration was used.

**Table 4:** Lipopeptide isoforms identified by MALDI-TOF mass spectrometry.

Lipopeptide	[M + Na]^+^ (*m*/*z*)	Isoforms
Pumilacidin	1030.6	C13 [Val7]
	1044.7	C14 [Val7], C13 [Leu/Ile7]
	1058.7	C15 [Val7] (pumilacidin B) ^a^, C14 [Leu/Ile7]
	1072.7	C16 [Val7] (pumilacidin F/G) ^a^, C15 [Leu/Ile7] (pumilacidin A) ^a^
	1086.7	C17 [Val7] (pumilacidin D) ^a^, C16 [Leu/Ile7] (pumilacidin E) ^a^
	1100.7	C18 [Val7], C17 [Leu/Ile7] (pumilacidin C) ^a^

^a^ See reference [15] for the pumilacidin nomenclature.

**Table 5:** Genes and putative proteins involved in surfactin/pumilacidin synthesis in *B. pumilus* SF214, *B. pumilus* SAFR-032, and *B. safensis* U17-1.

Genes	Putative Encoded Protein	Protein Identity (%) ^a^		
		*B. pumilus* SF214 vs. *B. pumilus* SAFR-032	*B. pumilus* SF214 vs. *B. safensis* U17-1	*B. pumilus* SAFR-032 vs. *B. safensis* U17-1
*srfAA*	surfactin synthase subunit 1	95	91	90
*srfAB*	surfactin synthase subunit 2	94	91	90
*srfAC*	surfactin synthase subunit 3	95	93	91
*orfX*	nonribosomal peptide synthetase	94	91	89
*orfY*	nonribosomal peptide synthetase	94	92	90
*srfAD*	surfactin synthase thioesterase subunit	94	89	87
*1*	hypothetical protein	-	87	-
*2*	hypothetical protein	-	89	-
*3*	hypothetical protein	-	90	-
*4*	hypothetical protein	-	84	-
*5*	hypothetical protein	-	90	-
*ycxC*	transporter	96	93	92
*ycxD*	transcriptional regulator	95	93	93
*sfp*	4′-phosphopantetheinyl transferase	94	88	88

^a^ The symbol “-” indicates the absence of the protein in *B. pumilus* SAFR-032.

## Supplementary Materials

The following are available online at http://www.mdpi.com/1660-3397/16/6/180/s1, Table S1: Marine isolated strains of the *Bacillus* genus, Table S2: Antimicrobial plate assay with 20 mL of filter-sterilized supernatant of SF214, Table S3: GenBank accession numbers^a^/locus tag^b^ for the sequences of SF214 and of closely related type strains of *Bacillus* species used in this study^a^.
