# Supplementary material for: A Marine Isolate of Bacillus pumilus Secretes a Pumilacidin Active against Staphylococcus aureus
Source: Mar Drugs. 2018 May 24;16(6):180. doi: 10.3390/md16060180 (PMC6025400; doi:10.3390/md16060180)
Supplement: Supplementary file 1 [file marinedrugs-16-00180-s001.pdf]

Table S1. Marine isolated strains of the *Bacillus* genus.

Strain ID	Species	Reference
SF200A	*Bacillus altitudinis*	[17]
SF203	*Bacillus firmus*	Laboratory collection
SF204	*Bacillus aquimaris*	[17]
SF206	*Bacillus pumilus*	Laboratory collection
SF208	*Bacillus altitudinis*	[17]
SF213	*Bacillus firmus*	Laboratory collection
SF214	*Bacillus pumilus*	[17]
SF216	*Bacillus pumilus*	Laboratory collection
SF217	*Bacillus firmus*	Laboratory collection
SF221	*Bacillus altitudinis*	[17]
SF222	*Bacillus marisflavi*	[17]
SF223	*Bacillus aquimaris*	[17]
SF224	*Bacillus pumilus*	Laboratory collection
SF241	*Bacillus firmus*	[17]
SF242	*Bacillus cibi*	[17]

Table S2. Antimicrobial plate assay with 20 ml of filter-sterilized supernatant of SF214.

Hosts	Supernatant activity of SF214[a]
Streptococcus faecalis ATCC 33186	0
Bacillus megaterium QMB1551	0
Staphylococcus aureus ATCC 6538	10
Listeria monocytogenes ATCC 7644	10
Mycobacterium smegmatis MC-2 155	0
Enterococcus faecalis ATCC 29212	0
Citrobacter freundii ATCC 43864	0
Pseudomonas fluorescens ATCC 13525	0
Shigella sonnei ATCC 25931	0
Escherichia coli ATCC 25922	0
Salmonella enterica typh. ATCC 14028	0

[a]Diameter (mm) of the inhibition halo in plate assays (Experimental section).

Table S3. GenBank accession numbers[a] of genome sequences and locus tags[b] of 16S and *gyrB* genes of the indicated bacterial strains.

	**Genome**[a]	**16S**[b]	***gyrB***[b]
Bacillus pumilus SF214	PRJNA290581	SF214_6rRNA16S	SF214_1687
Bacillus subtilis 6051-HGW	NC_020507.1	BSU6051_RS00040	BSU6051_RS00030
Bacillus amyloliquefaciens LL3	NC_017190.1	LL3_r0001	LL3_00006
Bacillus licheniformis DSM 13	NC_006322.1	BLI_RS00040	BLI_RS00030
Bacillus pumilus SH-B9	NZ_CP011007.1	UP12_RS00040	UP12_RS00030
Bacillus pumilus SAFR-032	NC_009848.4	BPUM_RS00040	BPUM_RS00030
Bacillus pumilus PDSLzg-1	NZ_CP016784.1	BEN31_RS02295	BEN31_RS15745
Bacillus safensis U41	NZ_CP015610.1	BSA41_RS00985	BSA41_RS17530
Bacillus safensis U17-1	NZ_CP015611.1	BSA171_RS00005	BSA171_RS00540
Bacillus safensis KCTC	NZ_CP018197.1	BSL056_RS00040	BSL056_RS00030
Bacillus cereus ATCC10987	NC_003909.8	BCE_RS00035	BCE_RS00025
Bacillus licheniformis ATCC9789	NZ_CP023729.1	CPQ91_RS00040	CPQ91_RS00030
Bacillus safensis BRM1	NZ_CP018100.1	BRL64_RS00040	BRL64_RS00030
